# Annotations

**Published:** 1894-03-03

**Authors:** 


### ANNOTATIONS.

### Massage in Edinburgh.

EDINBURGH has the advantage of being small as compared with London, and of containing among its inhabitants a large proportion of cultured persons and persons of character. Public opinion there has great restraining power. Massage, which has not always kept itself so entirely clean as it might have done in the South, is in excellent case in the Northern metropolis. The doctors who practise it, and the masseurs and masseuses who carry out the manipulations, seem all to be men and women of worth and responsible position. This is as it should be. There can be no doubt whatever in the minds of physicians of experience that the methods of the masseur constitute a real gain to therapeutics. It is, therefore, in all respects desirable that the art of massage should be kept free from the taint of quackery. Dr. Keiller, the son of a former President of the Edinburgh College of Physicians, and Dr. J. H. A. Laing, both practice this specialty, and, we believe, with much success. There are besides quite a considerable number of masseurs and masseuses, among whom Mr. P. J. Cusack has obtained a leading place. Mr. Cusack, who is a man of considerable all-round capacity, has started small classes for the training of men and women in the art of massage. In these he has, we believe, had decided success. Well-known physicians in Edinburgh have tested his pupils, and find them possessed of competent knowledge of the general duties of nurses, together with adequate practical capacity in manipulation. It must be a satisfaction to all right-minded persons to know that an art which so conspicuously lends itself to knavery, and which yet is of such very great service, is kept sweet and wholesome in Edinburgh.

### The Net Gain of the Chloroform Commission.

IT is not every doctor who can tell us what is the exact time of day on the clock of medicine even though he may see the dial of the clock staring him full in the face. In other words, many a medical man is twenty, thirty, or forty years behind the times even in the all-important art of mitigating human suffering and saving human life. But clearly this ought not so to be. There are not wanting many indications of a painful kind to show us that some physicians and surgeons of considerable standing have failed to fully realise, and to incorporate with their art, the important conclusions of the Hyderabad Chloroform Commission. Does chloroform kill by arresting the action of the heart. "No! absolutely and certainly no!" say the Hyderabad Commissioners. The healthy heart may therefore be neglected when chloroform is administered. But how then does chloroform kill? This question also the Commission has answered; and the answer is of the greatest conceivable daily importance in the practice of those who use anæsthetics. Chloroform kills by checking the respiration. But how then are we in practice to avoid checking the respiration? The answer is that we must avoid it; and the avoidance is to be effected by ensuring the free admixture of atmospheric air with the chloroform during its administration. Those inhalers which allow the patient to inhale the anæsthetic alone without the admixture of air are death-traps. It does not require anything but reasonable common sense to enable us to recognise, moreover, that some patients reach the suffocation point much more quickly than others. These should surely have the protection of a larger admixture of air and a slower administration. The practical conclusion then is that an anæsthetic, at any rate, if it be chloroform, should be administered slowly, not quickly; that the pace of administration must be regulated by the capacity for respiring adulterated air; that natural breathing and the evidences of adequate oxygenation of the blood, or otherwise, are the physiological indicators which should guide the administrator. If due care be taken to administer chloroform with an adequate admixture of air in each case, and to provide against the loss of animal heat which the anæsthetic entails, deaths from chloroform should be absolutely unknown. The Prime Minister of Hyderabad has done well, in his letter addressed to Drs. Gaskell, Shore, Hare, and Thornton, to remind the world of the excellent services rendered to medicine and humanity by the Chloroform Commission.

### Aids to Longevity.

THE philosopher may balance the advantages and disadvantages of long life, and may decide in favour of a short time of human existence. But it is clearly a prime part of the business of the physician to make life as long as possible, and as comfortable. There are two sorts of pressure which tend to shorten life, blood-pressure within and atmospheric pressure without. This latter is a specially important factor in a humid climate like our own. In advancing age the circulation of the blood and lymph tends to become slow, and the enfeebled heart finds its embarrassments increased by this condition. Especially do the more vascular organs, such as the lungs, the liver, and the kidneys put skids on the wheels of the blood circulation Plainly then an important condition of cardiac easement, and therefore of life-prolongation, is the maintenance of an uncongested state of lung, liver, and kidney. Thus are internal pressures relieved, and thus is cardiac energy conserved. Of almost equal importance, at any rate in Great Britain, is the question of atmospheric pressure and moisture to aged persons. Situations which are at once low lying and damp give, of course, a maximum of atmospheric pressure. Such pressure weighs down at a single stroke body, mind, and life. The difference to aged persons between living at the sea level, and living five hundred feet above it, between living in a moist atmosphere and living in a dry one, is sometimes quite incalculable. Not seldom life may be lengthened by five, or even ten years by living in an atmosphere which is both light and dry. These physiological considerations are commended to the aged and to the physicians of the aged. Whilst physiological explorers are busy in the laboratory, clinicians must not imagine that new discoveries can be applied in practice without constant and intelligent effort on their part. Knowledge, like freedom, "filters slowly down," but there is no objection to a little artificial acceleration of the pace.

